# The therapeutic potential of ethical dilemma resolution: A hypothesis

**DOI:** 10.1177/10398562241285976

**Published:** 2024-09-25

**Authors:** Laalithya Konduru, Simranjeet Singh Dahia, Gargi Kothari-Speakman

**Affiliations:** Adelaide, Australia; Dhanbad, India; Adelaide, Australia; Chennai, India; Chennai, India

Dear Editor,

Konduru and Das’s work,^1^ showing how Eastern philosophical principles can be applied to ethical dilemmas in end-of-life care, generated a thought-provoking post-publication discussion where a hypothesis emerged: resolving ethical dilemmas could function as a personalized mental health intervention.^2^ Empirical evidence suggests that ethical dilemmas represent a significant source of mental and emotional distress, exerting a tangible toll on individuals’ psychological well-being.^3^

By engaging in a structured examination of competing considerations, individuals can arrive at decisions that are ethically sound and personally meaningful. This structured approach, involving fact finding regarding the case, identification of the ethical dilemmas, researching precedents, enumerating the options, and formulating a plan of action, promotes clarity and conflict resolution. We hypothesize that this may ameliorate the psychological impact of moral uncertainty.

Consultation with ethics committees can be beneficial for the well-being of both medical practitioners and patients.^4^ Medical ethics committees must comprise ethicists, philosophers, theologians, legal experts, the clinical teams, and the patients themselves to ensure that ethical decisions are informed by diverse perspectives. Engaging with these committees can broaden the perspective and provide guidance when faced with complex decisions, potentially leading to more informed and balanced outcomes. In our institution, when an ethical dilemma arises, those seeking resolution can send a referral letter to the ethics committee, similar to how one would request a consultation with a medical specialist outside the home team. Upon receiving the referral, a member of the ethics committee arrives to conduct a consult, gathering all the facts of the case and reporting back to the committee. The committee then invites the stakeholders (patients, family members, other medical practitioners, hospital management, etc.) of the case to a meeting (or a series of meetings) to engage in the structured ethical dilemma resolution process. The plan is actioned by a designated stakeholder. Other models of ethics consultations have been reviewed in the literature.^5^

The process of ethical decision-making is inherently tailored to the unique circumstances, values, and potential consequences pertinent to each individual. By considering these personalized factors, individuals can navigate ethical dilemmas in a manner that resonates with their personal ethos and aspirations, fostering a sense of agency and empowerment.

Despite the intuitive appeal of utilizing ethical dilemma resolution as an intervention to alleviate mental distress, empirical research directly assessing its impact on mental health outcomes remains scarce. Consequently, rigorous empirical studies substantiating the mental health benefits of structured ethical decision-making are required. There is also a need to delineate whether it is the process of structured decision-making or the resolution of the ethical dilemma that contributes to alleviating mental distress. Such evidence would not only bolster the argument for considering ethical interventions as personalized mental health interventions but also pave the way for informed and effective interventions in clinical practice.

In conclusion, the hypothesis positing that resolving ethical dilemmas can serve as a mental health intervention offers a promising avenue for further research and has the potential to enhance our understanding of the complex interplay between ethics and mental well-being.
